# The double-edged sword of early antibiotic exposure in extremely preterm infants: implications for necrotizing enterocolitis and growth faltering

**DOI:** 10.1038/s41390-025-04088-9

**Published:** 2025-06-20

**Authors:** Jonathan A. Berken, Maria C. Rico, Joseph H. Chou, Juan D. Matute

**Affiliations:** 1 Department of Pediatrics, Perelman School of Medicine at the University of Pennsylvania, Philadelphia, PA USA; 2 Division of Neonatology, Department of Pediatrics, The Children’s Hospital of Philadelphia, Philadelphia, PA USA; 3 Division of Gastroenterology, Hepatology and Nutrition, Department of Pediatrics, The Children’s Hospital of Philadelphia, Philadelphia, PA USA; 4 Division of Newborn Medicine, Department of Pediatrics, Mass General Brigham, Harvard Medical School, Boston, MA USA; 5 Institute for Immunology and Immune Health, Perelman School of Medicine at the University of Pennsylvania, Philadelphia, PA USA

Despite the growing emphasis on antibiotic stewardship in the Neonatal Intensive Care Unit (NICU), infants born before 28 weeks gestation or weighing less than 1000 grams at birth remain disproportionately exposed to antibiotics, both prenatally and during the first postnatal week.^1,2^ This early and often prolonged antibiotic exposure significantly influences the delicate process of intestinal microbial colonization,^3,4^ which has been increasingly implicated in the pathogenesis of necrotizing enterocolitis (NEC)^3^ and growth faltering (GF).^4^

Strobel et al., in a secondary analysis of the Preterm Erythropoietin Neuroprotection Trial (PENUT), investigated how maternal perinatal and early neonatal antibiotic exposure influences NEC and GF in infants born before 28 weeks of gestation (extremely preterm infants).^5^ Their findings underscore the widespread use of early antibiotics. In fact, 90% of infants in the Strobel et al. analysis received antimicrobials, mirroring prior studies.^2^ They report that each additional day of antibiotic use in the first week of life increased the adjusted hazard ratio (aHR) for NEC. When 5 or more days of antibiotics were administered in the first week of life, the aHR was 1.95.^5^ Additionally, compared to infants exposed to 0–2 days of antibiotics, newborns who received more than 2 days of treatment in the first week exhibited a greater susceptibility to GF across all analyzed anthropometric measures, including weight, length, and head circumference (HC).^5^ Yet, the role of maternal antibiotic exposure during the birth admission painted a more nuanced picture. Unexpectedly, peripartum antibiotic use was associated with a reduced odds ratio (OR) of HC GF, with no effects on overall GF or NEC.^5^ These findings add another layer to the complex interplay between antibiotic exposure and neonatal outcomes.

Strobel et al. leveraged a large, contemporary, multi-center dataset, analyzing 891 infants for NEC and 828 for growth outcomes.^5^ Their rigorous methodology, including comprehensive multivariable adjustments, enhances the internal validity of their findings. Additionally, the study’s multi-site design promotes external generalizability across diverse NICU settings. The authors’ use of widely accepted clinical definitions, such as Bell’s stage ≥ IIA for NEC, the most applied research definition worldwide,^6^ and a decrease in weight, length, or HC z-score greater than 0.8 between birth and discharge for GF as defined by the Academy of Nutrition and Dietetics,^7^ ensures that their reported outcomes possess real-world clinical relevance.

Some limitations intrinsic to secondary analyses remain, which are appropriately acknowledged by the authors. The study lacked specificity regarding antibiotic types, limiting the ability to determine whether certain antibiotics differentially affect the host’s susceptibility to NEC and GF. Additionally, critical variables influencing NEC and growth—such as enteral feeding type, duration of nil per os, and reliance on parenteral nutrition—were not available, making it challenging to fully disentangle the effects of antibiotics from other care practices. Unexpected cohort characteristics, including a lower proportion of small-for-gestational-age infants in the no-antibiotic group and the absence of a clear gestational age-NEC relationship, further deviate from established epidemiologic patterns.^8^ Moreover, while multivariable adjustment is essential to minimize confounding, it can also diminish statistical power, potentially obscuring subtle associations.^9^ Finally, the study did not account for key clinical factors such as the type (e.g., dopamine, dobutamine, or epinephrine), dose, and duration of vasopressor use during the first week. These factors may alter antibiotic pharmacokinetics and clearance by variably affecting cardiac output and renal blood flow,^10^ while also directly influencing the microbiome by promoting bacterial proliferation,^11^ thereby introducing an additional layer of potential unmeasured confounding.

The debate over maternal prenatal antibiotic exposure just before birth and its link to NEC remains far from settled. Prior studies have suggested that certain antibiotics may heighten NEC risk, while others indicate a protective effect or no impact at all.^12–36^ Weintraub et al. reported that peripartum ampicillin exposure was associated with an increased risk of NEC.^12^ Kenyon et al., in a meta-analysis of 11 randomized clinical trials (RCT) including 6229 infants with preterm (<37 weeks gestation) rupture of membranes, found that NEC incidence was overall unchanged with prenatal antibiotics, with the exception of amoxicillin–clavulanate that increased NEC.^13^ In contrast, Reed et al. suggested that antibiotics within 72 hours before delivery might actually reduce NEC incidence.^14^ Strobel et al., however, did not identify a significant association between prenatal antibiotic exposure and NEC risk in extremely preterm infants,^5^ raising the possibility that the relationship is not uniform. Antibiotic selection, timing of administration, and the underlying clinical context may all critically shape the risk of NEC, complicating efforts to draw clear conclusions.

**Table 1:** Impact of early postnatal antibiotic exposure on GF and NEC risk*

	Study	Design	Population (GA at birth)	# of Infants	Antibiotic Exposure	Findings
**GF**	Strobel et al., (2025)^5^	Secondary analysis of RCT	< 28 weeks	828	First week of life	↑
	Zhu et al., (2023)^37^	Cohort	< 32 weeks	1834	First week of life: varying duration(≤ 4 days or > 4 days)	↑
	Reid et al., (2019)^57^	Cohort	30-32 6/7	431	First week of life	↔
**NEC**	Strobel et al., (2025)^5^	Secondary analysis of RCT	< 28 weeks	891	First week of life	↑
	Fillistorf et al., (2025)^61^	Cohort	< 32 weeks	1398	First week of life	↔
	Zhu et al., (2025)^62^	Cohort	< 34 weeks	24,926	First 3 days of life	↔
	Köstlin-Gille et al., (2023)^42^	Cohort	VLBW < 1500g (mean 29 weeks)	1762	First week of life: varying duration (1-2, 3-6, or >7 days after birth)	↑
	Vatne et al., (2023)^38^	Cohort	< 32 weeks	4932	First week of life	↑
	Zhu et al., (2023)^37^	Cohort	< 32 weeks	1834	First week of life: varying duration (1–4 days, >4 days)	↑
	Letouzey et al., (2022)^43^	Cohort	22-32	648	Day 0 or 1 of life	↔
	Dierikx et al., (2022)^47^	Cohort	< 30 weeks	1259	Before or after 3 days of life	↓
	Chen et al., (2022)^63^	Cohort	VLBW infants	132	First 14 days of life	↑
	Ruoss et al. (2021)^60^	RCT	< 33 weeks	55	< 2 days versus > 2 days; antibiotic selection at clinician discretion	↔
	Li et al., (2020)^8^	Cohort	VLBW infants (~30 weeks)	2831	First 3 days of life	↓
	Ting et al., (2019)^64^	Cohort	27-30 weeks	14,207	First week of life: varying duration (0, 1–3, 4–7 days)	↔
	Greenberg et al., (2019)^65^	Cohort	22–28 weeks gestation with birthweight 401–1000 g	5730	First 3 days of life: varying duration (0-4 days, ≥ 5 days)	↔
	Fajardo et al., (2019)^66^	Cohort	< 1250 g (~27 weeks)	620	First 14 days of life	↔
	Esmaeilizand et al., (2018)^39^	Case–control	< 29 weeks	4510	First week of life: varying duration (0-4 days, > 5 days)	↑
	Berkhout et al., (2018)^46^	Case-control	< 32 weeks	843	First day of life	↓
	Alexander et al., (2011)^41^	Case-control	~28 weeks	372	First 10 days of life: varying duration	↑
	Kuppala et al., (2011)^44^	Cohort	VLBW infants < 1500 g; < 32 weeks	365	First week of life: varying duration (1-2, > 5 days)	↔
	Tagare et al., (2010)^59^	RCT	< 37 weeks	140	First 5 days of life: IV amoxicillin-clavulanate and amikacin	↔
	Cotten et al., (2009)^40^	Cohort	Birthweight 400-1000 g (~26 weeks)	4039	First week of life: varying duration	↑
	Krediet et al., (2003)^48^	Case-control	VLBW infants (~29 weeks)	3602	First 2 days of life	↓
	Siu et al., (1998)^49^	RCT	VLBW infants	140	First 2 days of life: Oral vancomycin	↓
	Harms et al., (1995)^67^	RCT	Median 29 weeks; median birthweight ~1200 g	148	First week of life: Prophylactic IV amoxicillin upon central venous catheter insertion (~2-3 days of life)	↔
	Rowley et al., (1978)^68^	RCT	Preterm infants	100	First week of life: Oral Gentamicin	↔
	Boyle et al., (1978)^69^	RCT	< 36 weeks and < 2250 g (mean GA 32 weeks)	99	First week of life: Oral kanamycin (some also received parenteral antibiotics)	↔
	Grylack et al., (1978)^51^	RCT	High-risk premature neonates	42	First week of life: Oral gentamicin	↓
	Egan et al., (1976)^50^	RCT	Infants with birthweight <1500 g who survived to start enteral feeds	75	First week of life: Oral kanamycin, 15 mg/kg/day	↓

*All studies in this table were peer-reviewed and included premature infants with and without antibiotic exposure in the first week of life.

**↑** = increased risk of GF or NEC;

**↓** = decreased risk of NEC;

**↔** = no significant difference or change observed

Gestational age = GA

Note: Symbols indicate directional trends reported in individual studies; interpretation may vary by study design (e.g., RCT vs. cohort).

Neonatal antibiotic exposure during the first week of life has primarily been linked to an increased risk of NEC (Table 1).^37–42^ The risk appears to escalate with longer antibiotic courses, as demonstrated by Strobel et al., who observed a progressive increase in NEC hazard with additional days of antibiotic exposure.^5^ However, not all studies have yielded consistent results, and variations in study populations and inclusion criteria may help explain these discrepancies (Table 1). For example, Letouzey et al. and Kuppala et al. reported no association between antibiotic administration and NEC incidence in neonates 22–31 weeks and ≤32 weeks, respectively.^43,44^ In contrast, in a meta-analysis of 5 RCTs, including late preterm infants, Bury et al. reported that oral antibiotics protected from NEC.^45^ Similarly, Li et al. found that antibiotic use during the first 3 postnatal days was associated with a lower incidence of NEC, differing from the findings of Strobel et al.^5,8^ Notably, Strobel et al. focused specifically on extremely preterm newborns,^5^ whereas Bury et al. included infants ≤37 weeks gestation and <2500 g and Li et al. examined a broader cohort of very low birth weight (VLBW) infants.^8,45^ Several other studies have reported a protective effect of postnatal antibiotics on NEC incidence.^8,46–51^ However, it is important to recognize that these investigations did not solely focus on extremely low birth weight (ELBW) infants, unlike Strobel et al., who specifically examined this high-risk population with distinct baseline risks and susceptibilities.^52^ These conflicting findings highlight the challenge of balancing early infection management with the potential for unintended harm and underscore the importance of considering population differences when interpreting results.

The impact of maternal antibiotic exposure on GF in preterm infants before discharge is gaining attention.^53,54^ Previous retrospective cohort studies found no significant changes in head circumference, length, or weight from birth to discharge when comparing preterm infants exposed and not exposed to prenatal antibiotics.^53,54^ However, it is important to note that only 145 patients were assessed for HC and length.^54^ In contrast, Strobel et al., in a larger cohort of extremely preterm infants, observed that prenatal antimicrobial exposure was associated with reduced OR of GF, but had no significant effect on weight or length gain.^5^ This raises the possibility that prenatal antibiotic exposure may exert a more selective influence on neurodevelopment rather than overall somatic growth.

The association between neonatal antibiotic exposure in preterm infants and GF has become increasingly evident (Table 1). While earlier studies primarily examined term infants,^55,56^ Strobel et al. extended this investigation to extremely preterm neonates, demonstrating that antibiotic exposure for more than 2 days significantly increased the likelihood of weight, length, and HC GF.^5^ Their findings build on prior reports by Uzan-Yulzari et al. and Kamphorst et al.^55,56^ but uniquely emphasize this risk in extremely preterm infants.^5^ Nonetheless, the evidence remains mixed. Strobel et al. observed that antibiotic courses of 3–4 and 5–7 days were associated with increased GF across multiple growth indices.^5^ Prior retrospective very preterm cohorts have found either a decrease^37^ or no change^57^ in growth velocity after exposure to antibiotics during the first-week of life. Notably, no study to date has demonstrated a protective effect of early neonatal antibiotic exposure on growth outcomes—suggesting that, at best, antibiotics have a neutral effect on growth, and at worst, they may impede it.

The findings of Strobel et al. add to the growing literature on this topic^5^ as the NICU Antibiotics and Outcomes (NANO) trial advances.^58^ This prospective, multicenter randomized controlled trial aims to clarify the long-term effects of early-life antibiotic exposure on NEC, GF, and neurodevelopmental outcomes. By integrating detailed data on antibiotic use, feeding practices, and microbiome composition, the NANO trial seeks to disentangle the intricate balance between infection control and unintended harm, providing critical insights to inform NICU antibiotic stewardship. Notably, previous RCTs on the impact of early life postnatal antibiotics, that include NEC as an outcome, provide blueprints for larger investigations into adverse outcomes following antibiotic exposure in preterm infants.^59,60^ While these RCTs were not specifically powered for NEC, their key contributions lie in their methodological framework, paving the way for more extensive studies like the NANO trial.

What we can conclude thus far from the available evidence is that antibiotic use in the care of extremely preterm infants remains a double-edged sword, often lifesaving in the context of infection, yet carrying the inherent risk of untoward consequences. The findings by Strobel et al. underscore the critical importance of antibiotic stewardship in the NICU, where every decision holds the potential to shape the developmental trajectory of our patients. As we await the results of the NANO trial, these findings further emphasize the need for judicious and evidence-based antibiotic use to balance immediate survival with optimal long-term health outcomes.
